# The Complete Genome Sequence of *Proteus mirabilis* Strain BB2000 Reveals Differences from the *P. mirabilis* Reference Strain

**DOI:** 10.1128/genomeA.00024-13

**Published:** 2013-09-05

**Authors:** Nora L. Sullivan, Alecia N. Septer, Andrew T. Fields, Larissa M. Wenren, Karine A. Gibbs

**Affiliations:** Department of Molecular and Cellular Biology, Harvard University, Cambridge, Massachusetts, USA

## Abstract

We announce the complete genome sequence for *Proteus mirabilis* strain BB2000, a model system for self recognition. This opportunistic pathogen contains a single, circular chromosome (3,846,754 bp). Comparisons between this genome and that of strain HI4320 reveal genetic variations corresponding to previously unknown physiological and self-recognition differences.

## GENOME ANNOUNCEMENT

The gut commensal bacterium *Proteus mirabilis* is the primary cause of urinary tract infections in patients with long-term indwelling catheters (1–4). Interestingly, migrating colonies of *P. mirabilis* cells can distinguish self from non-self: a visible boundary forms at the interface between two genetically distinct colonies, while two genetically identical populations merge together (5). The genetic determinants of this self-recognition behavior, first identified in *P. mirabilis* strain BB2000, included self-identity genes containing numerous interstrain nucleotide polymorphisms and suggested that additional genetic differences between strains are likely (6). To date, only the genome of *P. mirabilis* strain HI4320 (NCBI accession no. NC_010554) has been completed (7). Here we report a second closed genome sequence, that of the genetically distinct strain, BB2000 (8).

BB2000 genomic DNA was isolated and sequenced using standard protocols. Briefly, DNA was isolated from cells cultured in modified LB using phenol/chloroform extraction and ethanol (9). Beckman Coulter Genomics (Danvers, MA) performed initial library preparation and sequencing using the Roche 454 platform. Illumina sequencing was used to confirm the 454 data and resolve stretches of unknown nucleotides; genomic DNA libraries were prepared according to the Illumina multiplexing sample preparation protocol and sequenced by the Harvard FAS Systems Biology Core using an Illumina HiSeq 2000. Illumina reads were assembled onto the 454 genomic data using Galaxy software (10). Genome closure was accomplished by amplifying across gaps using PCRs followed by Sanger sequencing performed by the Genewiz Corporation (South Plainfield, NJ).

The *P. mirabilis* BB2000 genome consists of a single chromosome (3,846,754 bp) with 38.6% G+C content. Potential coding sequences (CDS) were identified using the xBase annotation service, which predicted CDS regions using Glimmer (11), and assigned predicted protein products based on a direct comparison to the *P. mirabilis* HI4320 genome (12–16). CDS absent in the HI4320 genome were assigned “hypothetical protein” as the predicted product. Twenty-eight genes related to self-recognition (6, 17) were annotated manually using BLASTx (12) and the HMMER web interface (18). Sequence assembly and annotation were completed using Artemis software (19). The BB2000 genome encodes 3,457 potential CDS, of which 2,592 are assigned a putative function; the remaining 865 CDSs are classified as hypothetical proteins, with an additional 81 tRNA genes and 22 rRNA genes.

Comparison of the BB2000 genome to that of strain HI4320 (7) revealed 93% similarity between the chromosomes. The CDS unique to each genome include genes related to phage, toxin elements, and self recognition. The HI4320 genome encodes iron acquisition proteins that are absent in BB2000. Strain HI4320 also contains a plasmid (NCBI accession no. NC_010555.1) (7), and the HI4320 chromosome encodes a complete set of *tra* genes for conjugative transfer. No plasmid was identified in BB2000, nor does its genome encode *tra* genes or any HI4320 plasmid-borne genes. Further analysis of variations between *P. mirabilis* isolates will advance our understanding of the genetic determinants of pathogenicity and self-recognition.

### Nucleotide sequence accession number.

This *P. mirabilis* BB2000 complete genome sequence has been deposited in DDBJ/EMBL/GenBank under the accession no. CP004022. The version described in this paper is the first version.
